# Understanding suicidal pathways through the lens of a Dual-System Model of Suicidality in real-time: The potential of ecological momentary assessments

**DOI:** 10.3389/fpsyt.2022.899500

**Published:** 2022-11-28

**Authors:** Juliane Brüdern, Heide Glaesmer, Thomas Berger, Lena Spangenberg

**Affiliations:** ^1^Department of Medical Psychology and Medical Sociology, University of Leipzig, Leipzig, Germany; ^2^Department of Clinical Psychology and Psychotherapy, University of Bern, Bern, Switzerland

**Keywords:** dual-system model, suicidality, suicide attempt, ecological momentary assessment, impulsive system, reflective system

## Abstract

Within the ideation-to-action framework, existing theories of suicidal thoughts and behaviors (STBs) primarily focus on the linear progression of suicide risk. This, however, neglects growing evidence that many suicidal individuals do not experience their suicide attempt as a planned action, and in some instances deny even having experienced any suicidal thoughts. Furthermore, recent research has found that risk factors differ substantially between persons and that this is reflected in the variety of suicidal pathways. Considering the strong variability of STBs, new innovative theoretical concepts and assessment methods are needed to advance our understanding of multiple suicidal pathways. In this review, we apply a dual-system framework to suicidality, the Dual-System Model of Suicidality (DSMS), which accounts for two different systems of information processing and behavior. The first of these described is the reflective system, whereby STBs are viewed from a self-regulation perspective and thusly considered as maladaptive coping behavior to perceived discrepancies regarding important goals. Applying a feedback-based view such as this to STBs provides a deeper understanding into underlying psychological processes involved in the development of STBs. The second system described by the DSMS is the impulsive system. Here, STBs are seen as a maladaptive self-organizing pattern that gets activated in high-risk situations of acute stress, negative affect, and when resources of the reflective system are depleted. In this context, the DSMS is informed by a strength model of self-regulation, which assumes that self-regulation resources are limited, an aspect with important theoretical and clinical implications for the development of STBs. In order to demonstrate the theoretical and practical utility of the DSMS, this review draws mainly on studies using ecological momentary assessment (EMA), a technology that allows to investigate moment-to-moment changes in STBs, and is therefore well suited for capturing the complex interplay of self-regulatory and impulsive processes proposed by the DSMS. The application of a dual-system framework to suicide research represents an innovative and integrative approach for expanding our knowledge about fundamental processes and how their dynamics lead to STBs. The usefulness of the DSMS, implications for future suicide research with EMA, and clinical implications are discussed.

Suicide represents a major public health problem and accounts for 1.4% of all deaths globally. Every year, close to 700,000 people die by suicide, a loss that further profoundly impacts their families, society and the economy (1). Despite a considerable number of novel theories and models describing risk factors and underlying mechanisms leading to suicidal thoughts and behavior (STB), our ability to predict future suicidal behavior has not improved over the last five decades (2, 3).

## Limitations of current suicide research

The most recent generation of theories and models of STB can be referred to as “ideation-to-action” models (4, 5), which assume that suicide risk exists on a continuum that begins with passive suicidal ideation and progresses to active suicidal ideation, planning behavior, and eventually results in suicidal behavior. Based on the continuum model, individuals with a higher expression of active suicidal thoughts are often considered higher risk and more likely to transition to suicidal behavior. Suicide theories belonging to the ideation-to-action framework (e.g., the Interpersonal Theory of Suicide [ITS, (6)], the Integrated motivational-volitional model of suicidal behavior [IMVM, (7)] aim to identify between-person-differences using a small set of psychological risk factors (e.g., perceived burdensomeness, entrapment), which, in theory, should reliably distinguish between suicide ideators and attempters. In our view, the assumptions of those theories have several limitations. First, these theories assume that a small set of specific clinical risk factors (e.g., perceived burdensomeness, entrapment) are necessary to create STBs and therefore propose simple pathways via the interaction of a small set of psychological factors that cause STBs (8). Exclusively focusing on a few isolated clinical psychological variables, however, neglects the investigation of underlying *basic psychological processes* that lead to STBs and thereby thwarts our chances of better understanding suicidality. Second, there is emerging evidence suggesting the existence of non-linear processes that lead to suicide attempts (9–11). In a recent study by Wastler et al. (12), 54% of the participants who had recently attempted suicide denied having had active suicidal thoughts leading up to that event, and 23% denied having had any recent suicidal thoughts at all. Some individuals with active suicidal thoughts also deny experiencing a passive desire to die, and up to two-thirds deny having made a plan before their attempt (12, 13). Furthermore, recent studies also indicate that suicidal ideation and associated risk factors highly fluctuate within hours and show significant temporal instability (14, 15). Some of these trajectories are characterized by sudden shifts from low-risk to high-risk states skipping intermediate steps of suicidal ideation and/or planning behavior (12, 16, 17). A growing body of research suggests that individuals experiencing such shifts show a stress reactivity pathway characterized by fleeting suicidal thoughts, cognitive and affective dysregulation, and impulsive, unplanned suicidal behavior (11, 18). As a consequence of the highly fluctuating nature of STBs, it seems reasonable to assume that self-report suicide risk scales are inappropriate for assessing rapid changes during a suicidal crisis and have only limited clinical utility for predicting future suicide attempts (19). Here, the use of real-time monitoring techniques like ecological momentary assessment (EMA) on mobile devices has become prevalent in suicide research as a method of assessing variables of interest and change processes over time (20). With EMA, specific items are measured several times a day following a scheduled signal from the device or around events of distress or specific thoughts and behaviors. Thus, EMA makes it possible to measure moment-to-moment micro processes of STBs as well as the contexts within which they occur in daily live without recall bias, and has the potential to capture fluctuations of risk factors over the course of weeks, days, or even hours (21). For example, variability of suicidal ideation assessed in real-time has predicted post-discharge suicide attempts 2 and 4 weeks after discharge (22).

Taken together, retrospective methods in suicide-related research have the potential for inaccuracy due to recall bias, which may be particularly pronounced for individuals with high levels of psychological strain. Moreover, retrospective methods do not allow for measuring temporal associations between relevant variables, nor do they offer the ability to test process theories or model contextual factors that may influence these processes. Accordingly, several studies have shown that the use of risk scales failed to predict future suicide attempts (23), and the UK National Institute of Clinical Excellence advices in its Self-harm Guideline against the use of risk scales for predicting suicide (24). In this context, EMA represents a valuable method for examining between- and, even more importantly, rapid change within-person processes and offers the opportunity to detect multiple pathways of suicidality.

## New directions of conceptualizing suicidality: The Dual-System Model of Suicidality

**FIGURE 1:**
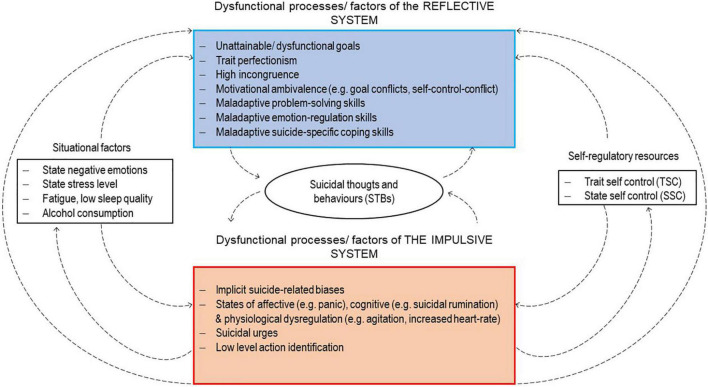
The Dual-System Model of Suicidality (DSMS).

In this article, we outline a Dual-System Model of Suicidality (DSMS, see Figure 1) that enables connecting basic psychological mechanisms of information processing to different suicidal pathways. Due to its manifold advantages outlined in the section above, we demonstrate how EMA can be utilized to investigate these concepts and account for the short-term effects involved in the development of a suicidal state.

### Dual-system models

Dual-system models are rooted in the fields of personality, social, and health psychology (25, 26). While a variety of dual-system models have been developed, they all share the general assumption that *two structurally different systems* of information processing underlie the generation of *impulsive* versus *goal-directed* forms of behavior, hence they term *two-mode-models*. Dual-regulation models assume that goal-directed behavior is driven by a *reflective* system, which is responsible for higher-order mental operations. These operations include executive functions like deliberate decision-making and evaluating, are slow in processing speed, and can be referred to as self-regulatory processes (27). From the perspective of dual-regulation models, impulses are generated by the *impulsive system*, are assumed to emerge from the activation of certain associative patterns, and can be defined as being implicit and bottom-up, since such processes emerge in the absence of conscious goals and operate in a connectionist manner (27). As mentioned above, recent suicide research has focused primarily on determinants and processes by which individuals take *reasoned action* to kill themselves, behavior which can be classified as a goal-directed action driven by the reflective system. Somewhat surprisingly, however, the influence of impulsive processes on suicidal behavior has received much less intention. In this article, we argue that a more complete model of suicidality should additionally integrate and specify impulsive influences on suicidal behavior. By applying a dual-system framework to suicide research, many relevant questions arise, for example, which reflective and impulsive processes determine suicidal behavior, and under which conditions do impulsive processes exercise a stronger influence on behavior in combination with a reduced influence of reflective processes. In the following section, we will first outline how suicide research has mainly integrated assumptions about the reflective system beyond conceptualizing suicidal behavior as a planned, goal-directed behavior.

### The influence of the reflective system on suicidal thoughts and behaviors

Carver and Scheier (26) have linked the **reflective system** of information processing to the *concept of self-regulation*. The self-regulation approach conceptualizes human behavior from a feedback-based view and the mechanism behind this view is a negative (discrepancy reducing) feedback loop. The loop includes personal goals and standards, which serve as reference points a person wishes to achieve. Moving toward a goal requires that a person identify their location on the relevant variable via a sensed input (the input function) and compare that sensed input to the reference point via a comparator. If the comparator recognizes a gap between their present location and their goal, adjustments are made to the output function. When a goal is focal, the process of moving toward it is presumed to be top-down, guided by that representation and managed and controlled by some sort of executive intentional process (26). According to the feedback-based view, such behavior is considered intentional and planned. Accordingly, the reflective system is also referred to as the “cool cognitive” system because it operates analytically and rationally. It is further assumed that operations of the reflective system are dependent on cognitive resources for the symbolic representation of, for example, deliberate evaluations that lead to reasoned actions and standards to restrain behavior in accordance with one’s goals (27, 28).

Recent theories of suicidality have already integrated *parts* of the self-regulation approach, although they do not explicitly refer to it. For example, the Interpersonal Theory of Suicide (ITS) (6) posits that suicidal thoughts are caused by two interpersonal constructs: thwarted belongingness and perceived burdensomeness. Translating this into terms of self-regulation, the comparator signals a discrepancy regarding an important interpersonal goal and this gap produces negative feelings in the form of e.g., thwarted belongingness and/or perceived burdensomeness. For example, a person is sitting alone on a park bench watching a family having a picnic. At this moment, the goal “Be part of a group” is activated and the person notices a discrepancy between their current situation and their goal. As a result, the person develops a feeling of thwarted belongingness, or in self-regulation terms, the comparator perceives a gap regarding an important goal. Current theories of suicidality suggest a range of cognitive-affective states, e.g., perceived burdensomeness and feelings of entrapment, that may serve as clinical risk factors for STBs (6). Findings of retrospective studies regarding these specific risk factors are heterogeneous (3, 29). Moreover, EMA studies have shown that these affective states show high fluctuations within persons and are not necessarily associated with short-term suicide ideation (15, 30). Therefore, findings support the assumption that suicide research should investigate more underlying processes instead of focusing on highly specific variables as proximal risk factors for STBs.

According to the self-regulation perspective, when a person detects a discrepancy they use self-regulation strategies in order to cope with it, and work to minimize the deviation. For example, the person on the park bench might call her husband to plan a dinner date. Thus, the discrepancy regarding the activated goal decreases, and feelings of thwarted belongingness are reduced. If this repeatedly fails, the discrepancy and its emotional side effects continue and suicidal thoughts may arise. There is emerging evidence that suicidal thoughts reduce negative emotions, an assumption referred to as the affect-regulation hypothesis (31). A current meta-analysis of EMA studies supports this hypothesis, having found that negative affect is generally higher before suicidal thoughts occur and is reduced following suicidal ideation (31). According to this framework, suicidal thoughts represent a self-regulation strategy, which can be viewed as a form of coping behavior for dealing with negative emotions. Moreover, if negative affect persists, ideation-to-action-models postulate that individuals *intentionally* begin to plan suicide, and ultimately apply a top-down controlled, goal-directed behavior with the conscious goal or intention of killing themselves, an assumption that aligns with the feedback-based view of the DSMS. This further implies that planning and suicidal behavior can be viewed as a self-regulation strategy or coping behavior, albeit maladaptive, which serves to cope with a discrepancy with regard to important goals or personal standards. In line with this assumption, findings suggest that planned suicidal attempts were associated with a greater intent to die and were more lethal compared to impulsive suicide attempts (11).

Nevertheless, as clinicians in particular know, not all people have suicidal thoughts or attempt suicide when they feel depressed or stressed, leaving the question of which individuals develop STBs and which do not unanswered. In addition to contributing new evidence that multiple pathways for STB exist, the self-regulation approach also defines relevant variables for investigation that have received little or no attention to date of how STBs develop.

#### Self-regulation on the level of goals

Suicide research has spent considerable effort identifying negative cognitive-affective states (e.g., perceived burdensomeness, hopelessness) under which individuals develop STBs (5). During this process, identifying the sources of these aversive states was rather left behind, although this information might be essential for our understanding and treatment of patients experiencing STBs. To date, only Vohs and Baumeister (32) have explicitly referred to the concept of self-regulation involving the aspect of personal goals in this context. In their Self-regulatory Model of Suicide, they proposed suicide attempts are the result of a desire to alleviate aversive self-awareness: “The process starts with some event that produces or makes salient a discrepancy between one’s goals or expectations and one’s actual current state. Falling short of important personal standards triggers feelings of self-blame and creates doubts about the attainment of future goals, resulting in internal, global, and stable attributions for negative events.” Although goals are important determinants of our behavior, as Vohs and Baumeister pointed out in their theoretical considerations about suicide, we know almost nothing about the role of goals in self-regulation among suicidal individuals. What we do know is that striving for personal goals is an important source of subjective wellbeing (33). *Approach goals* are developed to satisfy basic needs (e.g., achievement, attachment, control, self-esteem), whereas *avoidance goals* are developed to prevent these basic needs from being frustrated. According to Grawe’s consistency theory, a goal structure presenting predominantly avoidance goals (e.g., avoid showing weakness) is strongly associated with psychopathology and poor wellbeing. It is assumed that avoidance goals are maladaptive because they hinder goal satisfaction, and consequently the satisfaction of basic needs. Grawe calls this state of unsatisfied goals “incongruence” (33), a term for the discrepancy between current achievements and personal goals with regard to the negative feedback loop. Incongruence produces negative affect and distress which are interpreted as warning signals by the comparator when standards are not met.

Recent suicide theories have mainly focused on frustrated needs regarding interpersonal goals and the resulting negative affective states (6, 7). It currently remains unknown, however, which specific goals trigger a sense of incongruence among suicidal individuals. Consequently, it limits suicide research if we only investigate emotions that result from frustrated interpersonal goals. This may be one reason why, for example, perceived burdensomeness is not a reliable risk factor for STBs (30, 34) while, by contrast, feelings of entrapment have been found to be a more reliable predictor for suicide attempts (35, 36). This could be due to the fact that entrapment might express a general feeling of failure that extends beyond interpersonal goals. Furthermore, Brüdern et al. (37) identified a goal structure shared by 17 suicide attempters, that is predominantly characterized by avoidance goals related to satisfying basic needs. It also became obvious that the suicide attempts served various avoidance goals such as “avoiding appearing weak” or “avoiding criticism.” Further research has shown that perfectionism is associated with suicidal ideation, indicating that individuals with STBs hold (excessively) high standards regarding their own person and in the interpersonal context (38, 39). A study by O’Connor et al. (40) found that difficulties with re-engaging in new goals predicted self-harm re-hospitalization 2 years after the index suicide attempt when disengagement from existing unattainable goals was low. Therefore, investigating goal characteristics and the underlying processes of goal engagement and disengagement reflects a promising direction for future EMA suicide research.

#### High-level vs. low-level identification of goals

In the context of goals, we next shortly refer to the assumptions of action-identification theory by Vallacher and Wegner (41). They propose that goals that guide action can vary regarding their level of identification. For example, “riding a bike” can be associated with a rather high-level identity or higher-order goal, like “seeing the neighborhood.” Low-level identities encompass more lower-level acts or lower-order goals like speeding up or crossing the street, although phenomenologically the person is doing the same thing. This concept might be especially relevant with regard to results of the study by Wastler et al. (12) showing that 31% of suicide attempters only experienced passive suicidal ideation before their suicide attempt. They named this the “passive suicidal ideation only” pathway to suicidal behavior in which individuals experienced thoughts like “I wish I could go to sleep and never wake up” or “I cannot imagine anyone being able to withstand this kind of pain.” In this context, Vohs and Baumeister (32) used the term cognitive deconstruction, which is marked by an orientation to the present, an awareness of concrete stimuli, and striving for lower-level goals at the cost of higher-order goals and a high-level identity. It is possible that individuals showing this pathway do not regulate their behavior according to higher-order goals or “plans” like “end my life” or “kill myself,” but rather act on a low-level identification characterized by low-level goals. An example of this would be “I’m going to take these pills in order to stop this unbearable pain right now” (low-level-identification) as opposed to, “I’m going to take these pills in order to die by suicide and disappear forever” (high-level identification). Operating in the low-level-identification modus might prevent a person from experiencing inconsistency in relation to higher-order goal conflicts (e.g., the goal of killing oneself might conflict with the goal of watching one’s children grow up and protecting them from emotional trouble) and thus might contribute to suicidal behavior being executed without interferences. This change in thinking is also demonstrated in a qualitative study with men who survived a suicide attempt (42).

#### Self-regulation on the level of the output function

A further reason, why not every person with goal discrepancies develops suicidal thoughts or crosses the threshold to suicidal behavior may be found in differences that exist on the output function level, or more colloquially, differences in an individual’s self-regulation strategies or coping behavior. Referring back to our example of the person on the park bench, he or she has several paths available to them for coping with feeling lonely. Depending on how the person deals with this situation, their mood might improve or worsen, potentially resulting in an activation of suicidal thoughts. If their emotion-regulation strategies are ineffective, then *self-regulation fails* and negative affect persists. As such, learning more about coping behavior in suicidal individuals could play a key role in understanding between-person differences in the context of suicidality as well as within-person differences. Correspondingly, a lack of knowledge about the within-person nature of coping behavior and associated variables prevents us from understanding why an individual is able to deal with strong suicidal thoughts on one day, but not another.

Existing EMA studies examining suicidal individuals’ coping patterns can be divided into two domains: *coping with negative life events and daily stressors* and *suicide-specific coping*. The first involves coping strategies for dealing with negative life events and daily stressors, whereas the latter involves coping strategies for dealing with suicidal thoughts and urges. Clearly distinguishing between the two is important, as strategies that are useful for dealing with daily stressors are not necessarily effective for dealing with suicidal thoughts and urges. Retrospective studies found that an avoidant and emotion-focused coping style in the presence of negative life-events and stressors was associated with increased STB, whereas a problem-solving-focused coping style and positive reframing have been found to be protective regarding STB (32, 43, 44). To the best of our knowledge, only two EMA studies exist that have investigated coping styles of suicidal individuals with negative stressors. The first study applied a six-day EMA design that examines different coping styles of suicidal adults for dealing with negative thoughts, which are viewed as daily stressors, and their prospective relationship with suicidal ideation (45). Results indicate that maladaptive thought control strategy use (worry and punishment) and rumination predicted suicidal ideation. In contrast, adaptive strategies (distraction, social control, and reappraisal) served as negative predictors for suicidal thoughts. The second EMA study investigated coping behavior of suicidal adolescents in reference to stressful situations (46), the findings of which suggested that suicidal adolescents used more coping relying on themselves than on others.

Regarding suicide-specific coping, Simon et al. (47) asked participants in an online survey which adaptive (e.g., distraction, positive self-talk) and maladaptive (e.g., alcohol and substance use) coping strategies they apply when experiencing suicidal thoughts. They found, for example, that almost 90% of participants used distraction, a method they experienced as moderately useful. In general, suicide attempters found many strategies to be less helpful than suicide ideators did.

Studies that have used EMA to examine suicide-specific coping longitudinally have so far mainly focused on “adaptive” strategies, recommended in a crisis plan for suicidal individuals. In a study carried out by Stanley et al. (48), they found that in a sample of participants with borderline personality disorder and suicidal ideation, only distraction/positive activity-oriented strategies (e.g., socializing, keeping busy) lowered the intensity of suicidal thoughts, whereas mindfulness-oriented strategies (e.g., self-soothing, sitting with feelings until they pass) did not decrease suicidal ideation in the short term. Al-Dajani et al. (49) investigated coping strategies with reference to suicidal urges in adolescents. They found that, on the within-person level, greater professional support seeking and perceptions of coping strategy effectivity on the previous day were associated with lower next-day suicidal urges. On the between-person level, adolescents who reported greater average use of cognitive strategies and personal support seeking from family/friends had lower daily suicidal urges.

#### Maladaptive self-regulation reduces self-regulatory resources

Muraven and Baumeister showed that self-regulatory resources are crucially limited and propose a *strength model of self-regulation* (50). They describe how self-regulatory resources can become depleted, a process referred to as *ego depletion* (51). Findings by Dang et al. (52) demonstrate an ego depletion effect by tracking how performance on a task that was demanding for the self-regulatory system reduced performance on a subsequent task, a phenomenon only found in the ego-depletion group. In addition to laboratory studies, ego depletion research has shown that individuals are less successful at exhibiting goal-directed behavior and restraining impulses if their self-regulatory resources are depleted. For example, participants who were assigned a thought-suppression task, which depletes self-regulatory resources, were less able to restrain their alcohol intake compared to a control group (53).

In our view, the strength model of self-regulation has far-reaching implications for suicide research. For example, when self-regulation is maladaptive and fails to downregulate emotions and suicidal ideation, further self-regulation is warranted, and resources become further depleted, leaving a person less and less able to manage suicidal urges. This assumption is also formulated in a conceptual paper by Bryan and colleagues (54) in which they describe change processes and the emergence of suicidal behavior based on the Fluid Vulnerability Theory of Suicide. The authors conclude that “the likelihood that suicidal behavior will emerge is significantly increased when an individual switches from a low risk state to a high-risk state *as a consequence of failed self-regulation*” (54). Put in self-regulation technical terms, when efforts on the output function level fail to reduce the discrepancy between the actual state and a desired goal, the person can either abandon or change the desired goal (e.g., change “avoid negative emotions” into “accept negative emotions”), or can further self-regulate until the discrepancy is diminished. This can be exhausting, especially when goals are unattainable or self-regulation strategies are ineffective. This implies that *maladaptive self-regulation makes a person vulnerable* to entering a state of ego depletion in which the influence of the reflective system is reduced and the influence of maladaptive impulsive suicidal processes increases, a situation we address in the following sections.

#### Trait and state self-control

In this context, we want to address a specific form of self-regulation, namely self-control. Sometimes, the terms “self-regulation” and “self-control” are used interchangeably (55). In this article, we use the term “self-regulation” more broadly to refer to the described processes of the feedback-loop, while “self-control” represents a discrete aspect of self-regulation defined as an individual’s capacity to actively override or inhibit impulses, suppress urges, and resist temptations (56), an ability that significantly contributes to successful self-regulation and goal-pursuit. Trait self-control (TSC) is viewed as the dispositional ability to override or inhibit undesired behavioral tendencies (such as impulses) and has been linked to positive outcomes including academic success, social relationships, and health. By contrast, poor TSC is associated with psychopathology, poor health, and financial instability (56). State self-control (SSC) refers to an individual’s momentary capacity to actively override or inhibit impulses, suppress urges, and resist temptations (57). From the perspective of the DSMS, the concept of SSC is especially relevant to STBs. As mentioned above, STBs can be viewed as a coping behavior that has gained a *positive hedonic value* through a person having experienced reduced negative emotions following STBs (31). At the same time, STBs can exist in contradiction with important personal goals in many suicidal individuals who experience motivational ambivalence regarding STBs and are motivated to restrain that behavior (58, 59). This implies that a negative-affect-decreasing pattern competes with personal goals (e.g., reasons for living) that exist in contradiction with this behavior resulting in a *self-control conflict*. When experiencing a self-control conflict, a person has to suppress or inhibit suicidal urges or *impulses* that can feel like a “craving” to give in to the negative-affect-decreasing pattern. While it makes sense to introduce the term “impulse” as it relates to STBs at this point, the related processes are explained more thoroughly in the following section “The influence of the impulsive system on STBs.” Suicidal impulses can be viewed as pattern of the impulsive system that has been established in order to cope with an aversive state in an effortless manner when self-regulatory resources are limited or depleted. According to the strength model, acts of self-control such as inhibiting and restraining suicidal impulses in order to pursue long-term goals consume energy and strength, reducing one’s capacity for further self-control (51). When self-regulatory resources are depleted, impulsive processes exercise a stronger influence on behavior while simultaneously reducing the impact of the reflective system (60). In the context of suicidality, when an individual experiences a self-control conflict such as dealing with suicidal thoughts and urges over a longer period of time self-control resources can become depleted and lead to a *self-control failure* in the form of a suicide attempt.

### The influence of the impulsive system on suicidal thoughts and behaviors

In contrast to self-regulatory processes, which are cognitively presented, monitored and shielded and therefore consume a lot of resources, impulsive processes operate in an effortless manner and at a fast processing speed (27, 61). In other words, impulsive processes are assumed to activate associated behavioral schemas regardless of whether cognitive resources are available at the moment or not (60). Therefore, in a state of reduced or depleted self-regulatory resources, impulsive processes exercise a stronger influence on behavior while simultaneously reducing the impact of the reflective system. From the perspective of dual-system models, impulses are assumed to be triggered in the **impulsive system** (27). Carver and Scheier (26) have linked the impulsive system of information processing to the *concept of self-organization*. Self-organized behavior is characterized by bottom-up processing and is a key feature of connectionist models, within which central executive controlling or directing processes do not exist. Consequently, unlike reflective processes, these processes are not characterized as being top-down or goal-directed. The dominant mechanism of the impulsive system is *reducing overall error* meaning that the system satisfies as many constraints as possible, *leading to minimal tension in the system*. Tension is defined as the sum of unsatisfied constraints of the system (62). Consequently, associative patterns, which can be viewed as processes of the impulsive system, emerge in a self-organized way and are created or strengthened through repeated co-activation of external stimuli, affective reactions, and associated behavioral tendencies (60). Most important, the impulsive system operates quickly in emotionally charged situations (when cognitive resources are limited and rational thinking is impaired) and with little effort, which is why it is also referred to as the “hot emotional system” (28). For example, during an aversive negative state, which presents as a state of high tension in the system, suicidal thoughts or, in the worst case, suicidal behavior, emerge bottom-up as a coherent pattern satisfying certain constraints and begin pulling toward reducing tension in the system (and perceived negative affect). Assumptions of connectionist models have already successfully been applied in simulating the emergence of suicidal ideation using the mindsponge mechanism (9) or network analysis (63), and other psychological problems [e.g., panic attacks, (64)] using computational models.

It is important to emphasize that, compared to goal-directed suicidal behavior driven by the reflective system, this behavior is not activated by a cognitively represented goal to attempt suicide, and therefore cannot be viewed as a reasoned action. Once an association pattern between STBs and tension reduction has been established, it can quickly be prompted when triggering conditions are present (16, 65). As such, STBs can be viewed as *impulses* whereby a prepotent pattern has been established in order to cope automatically (with little effort) with an aversive state signaled by the comparator.

#### Measuring implicit suicidal associations of the impulsive system

Behavioral assessment tools have been used in suicide research to measure implicit associative patterns such as *implicit cognitive attitudes toward death* and *suicide-specific attentional bias* in order to assess suicide risk beyond self-report. These procedures represent a new class of implicit measurement tools for measuring suicide-specific implicit processes and associations that run automatically and unconsciously. For example, the Death-Implicit-Association-Test (D-IAT) measures a death-identity bias, which has been found to predict lifetime and future risk of suicide attempt (66). A recent systematic review concluded that the D-IAT showed an adequate predictive power regarding its validity for future STBs, albeit with only a small number of studies (67). Another area of study focuses on attentional biases to suicide-related stimuli, most easily represented by a formulation of the Stroop Task with suicide-related stimuli. In the Suicide Stroop Task (SST) individuals are asked to name the color of suicide-related- positive- negative- or neutrally-valenced words. Initial results have demonstrated that increased interference for suicide-related words—indicating a suicide attentional bias—was associated with increased risk for suicide attempts at 6-month follow-ups and in the wake of recent suicide attempt (68). A recent meta-analysis, however, found poor classification accuracy, indicating that the SST performed near chance in distinguishing between suicide attempters and non-attempters (69). That notwithstanding, authors still consider the SST to be a useful tool in concept for measuring attentional biases providing that it be modified in some concrete ways.

#### Suicidal impulsive precursors

Although suicide-related implicit biases can also be viewed as a type of suicidal impulsive precursors, we now want to address precursors that are not exclusively unconscious. Suicidal impulsive precursors are characterized by states of affective dysregulation such as *panic and anxiety*, cognitive dysregulation such as rumination in general (45) and *suicidal rumination*, physical dysregulation such as high *agitation and hyperarousal* (16), which do not represent top-down, reflective operations controlled by the reflective system, but rather maladaptive self-organizing processes of the impulsive system signaling a state of *high overall error or high tension* due to a high level of incongruence.

When an individual is in a state of affective and cognitive dysregulation, they are highly stressed and *cognitive and control resources for reflective operations are reduced* resulting in a diminished capacity for higher-order mental operations such self-monitoring or exercising restraint in accordance with important long-term goals (32, 60). Under such conditions, the system is unstable and rapid state changes become very likely, including the emergence of suicidal behavior as an impulsive suicidal pattern for reducing high tension in the system (16, 54, 62, 65). These assumptions are in line with recent findings showing that individuals with impulsive suicidal behavior demonstrate having a stress reactivity pathway characterized by fleeting suicidal thoughts and cognitive and affective dysregulation (11, 18).

An increase of such impulsive processes as precursors of short-term suicidal behavior is also described as *Suicidal Crisis Syndrome (SCS)* by Galynker et al. (70). They define SCS as being a presuicidal acute risk state of cognitive and affective dysregulation featuring some of the aforementioned factors, for example, loss of cognitive control (e.g., ruminative flooding), affective disturbance (e.g., frantic anxiety), and hyperarousal (e.g., agitation). Currently, EMA research has yet to investigate SCS prospectively [an EMA study of our research group is under way, see (71)], but individuals who met SCS criteria were nearly seven times more likely to attempt suicide within 4–8 weeks following inpatient discharge (70).

With regard to suicidal impulsive precursors, we want to refer back to the Action Identification Theory (41) introduced in the self-regulation part of this article. A low-level identification was defined as an orientation to the present, an awareness of concrete stimuli, and striving for lower-level goals at the cost of higher-order goals and a high-level identity (32), phenomena which have been repeatedly linked to the operating modus of the impulsive system (62). This indicates that suicidal individuals operating in that modus are likely to be rather *low-level identifiers*, something that puts them at further risk of developing an impulsive suicidal pathway. By extension, they can be expected to experience “suicidal thoughts” on a rather low-level (e.g., “stop that pain”) as opposed to a high-level (e.g., “I wish I could die”). Moreover, when people are operating on a low-level identification, for example during a high-risk situation when self-regulatory resources are depleted, they have no access to higher-order adaptive goals that might inhibit a suicide attempt. As a result, impulsive suicidal behavior can be carried out although it might be in high conflict with personal higher-order goals. From this perspective, it becomes plausible that suicidal behavior could emerge in a person without their experiencing active suicidal thoughts, desires, or planning behavior at the time as a result of the impulsive system, which is associated with a low-level action identification, overriding their reflective system. This further implies that individuals might be capable of attempting suicide despite being highly ambivalent about it if they enter a mode of a low-level identification in which the suicide attempt becomes untethered from high-level identification processes.

### Situational conditions depleting self-regulatory resources in suicidal individuals

We will now cover certain *situational conditions* that can deplete self-regulatory resources and thus lead to the activation of maladaptive impulsive suicidal processes as well as weakening a person’s restraint again those impulses.

#### Sleep

Ego depletion research has shown that *fatigue* (72) and *poor sleep* (73) moderate the relationship between self-control demands and a reduced self-control capacity. Although there is no direct evidence that sleep problems influence an individual’s self-regulatory behavior or diminish their capacity for self-control during a suicidal crisis, recent EMA research has shown that poor sleep quality and short sleep duration were temporally associated with suicidal ideation (74). Furthermore, an EMA study (75) with high-risk adolescents has found that shorter-than-usual sleep duration predicted the presence and intensity of next-day suicidal ideation via heightened affective reactivity to negative interpersonal events, a finding in line with the DSMS. According to its assumptions, when a person has depleted self-regulatory resources due to sleep deprivation they are not able to downregulate negative affect via effective emotion-regulation strategies and that results in affective dysregulation. As mentioned above, states of affective dysregulation further deplete self-regulation resources, which, in turn, goes hand in hand with the impulsive system gaining influence over information-processing and behavior, a dynamic which might present as an increase of suicidal thoughts, urges, and (when self-regulation resources are completely depleted) behavior. Consistent with this perspective, Kleiman et al. (76) refer in a recent EMA study to “regulation depletion models” and found a moderating role of fatigue in the relationship between perceived stress and suicidal ideation. They conclude, that fatigue might have an influence on situational coping behavior meaning that on days with high perceived stress and fatigue, individual’s coping resources are diminished and this can result in increased suicidal ideation, an assumption that closely corresponds with the DSMS.

#### Alcohol intoxication

Research has shown that alcohol consumption narrows the perceptual focus to the most salient environmental cues, reinforces negative affect, and consequently, long-term goals drift out of focus and emotional behavior driven by the impulsive system becomes more apparent (60). Studies using self-report measures indicate that unplanned suicide attempts were associated with the amount of alcoholic drinks consumed daily regardless of diagnosis (77–79). In a military sample, alcohol use during the 24-h-period prior to a suicide attempt was significantly associated with impulsive suicidal behavior (80). These findings clearly indicate that the impulsive system’s influence increases when suicidal individuals consumed alcohol. According to the DSMS, suicidal individuals’ reflective information-processing abilities can be impaired by alcohol, leading, for example, to impaired coping behavior, affective dysregulation, and reduced access to adaptive long-term goals. Furthermore, alcohol consumption might lead to low SSC and a person losing their capacity to resist suicidal urges, resulting in a self-control failure in the form of impulsive suicidal behavior. To date, we have little knowledge about the underlying processes of how alcohol intake leads to impulsive suicidal behavior. We do know though that alcohol consumption is a maladaptive coping strategy that facilitates impulsive suicidal pathways in suicidal individuals, and that being the case, EMA studies integrating alcohol use as a variable need to be conducted among that vulnerable population (47).

#### Acute stress and negative affect

Not only do states of affective dysregulation deplete self-regulatory resources, negative affect and acute stress (signals of perceived incongruence) also generally impair functions of the reflective system. As mentioned above, the reflective system represents the “cool cognitive” system which is characterized by careful examination of competing goals, objectively weighing the pros and cons of decisions, reasoned actions, and monitoring goal-pursuit by inhibiting impulsive processes (62). Operations of the reflective system consume cognitive resources and in situations of acute stress or aversive affect when these resources are sparse the impulsive system begins to assume greater influence. This implies multiple negative consequences, for example, implicit suicidal biases having a greater impact on information-processing and behavior. For example, suicide ideators demonstrated significantly weaker implicit associations with life in a Death-IAT after a negative mood induction compared with associations before the mood induction indicating that in states of negative mood, impulsive processes become activated (81). Due to a negative-affect-decreasing experience with STBs, this pattern can quickly be activated by the impulsive system when a person is suffering under aversive stress and emotions. Because self-control capacity is low in emotionally charged situations (60), the person is not able to cope adequately with suicidal ideation and urges and the risk for suicidal behavior increase greatly. A recent EMA study (82) partially confirms this view by showing a positive association between NA and the number of days with suicidal thoughts 2 weeks after discharge and a positive association with post-discharge suicide attempt within 4 weeks after discharge.

### Avenues for future ecological momentary assessment research deriving from the Dual-System Model of Suicidality

**FIGURE 2:**
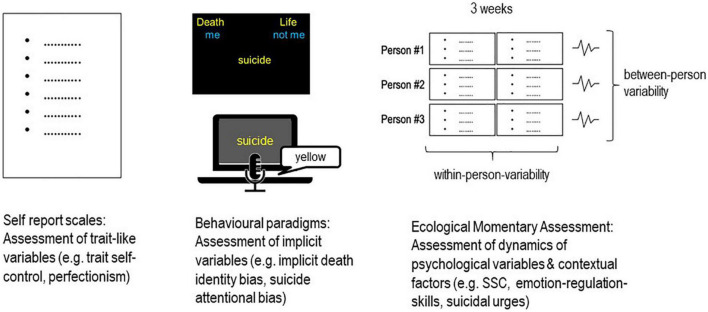
Research design integrating different assessment methods for investigating psychological variables and processes.

The DSMS offers a number of avenues for future research into the dynamics of the reflective and impulsive system in suicidal individuals (see Figure 2), including using the potential of EMA as a technology for capturing these dynamics in real time. First, we need more profound knowledge about basic within-person processes of goal attainment, perceived expectancies for success, and affect regulation regarding frustrated goals (high degree of incongruence). Future EMA studies should also involve the investigation of goal conflicts and its influences on affect and STB. This could help to identify critical states of within-person incongruence which are associated with high levels of negative affect and stress and therefore represent high-risk states for STBs.

Second, we need more EMA research investigating suicidal individuals’ daily coping behavior to better our understanding of maladaptive self-regulatory processes leading to STBs. On the one hand, future EMA research should focus on problem-solving skills and emotion-regulation strategies with regard to frustrated goals, needs, and associated negative affective states. In this context, a comparison with a *non-suicidal control group* is essential for learning more about the underlying basic processes related to suicide (and determining which processes are not related). On the other hand, future EMA studies should also investigate suicide-specific coping among more high-risk samples, for example suicidal individuals after discharge. They should further use a more naturalistic design by including adaptive and maladaptive coping strategies suicidal individuals use in their daily lives. This could help to identify maladaptive processes in which self-regulation fails and suicidal behavior might become more likely.

Third, TSC and SSC variables have yet to be examined in suicide research. In our opinion, the integration of self-control in suicide research is highly relevant because EMA studies could help to identify high-risk periods in which suicidal impulsive precursors become dominant, facilitate states of ego-depletion, and degrade an individuals ability to cope with negative affect and suicidal thoughts and urges. In such high-risk periods, which are likely to be characterized by an individual having low-state-self-control-capacity, it seems highly relevant to investigate how emotion-regulation strategies and suicide-specific coping behavior change compared to periods in which SSC is not reduced. For example, a recent EMA study in personality psychology investigated how self-control is associated with emotion-regulation and affective wellbeing in daily life (83). They found that TSC and SSC were associated with affective wellbeing in daily life, and that SSC was a mediator between adaptive emotion regulation and affective wellbeing, rendering emotion-regulation more arduous and contributing to lower levels of self-control capacity in the moment, thus impacting how people feel in daily life. EMA designs such as that presented by Wenzel et al. (83) offer great potential for suicide research investigating whether TSC and SSC are also associated with STBs and whether they moderate self-regulatory processes (e.g., emotion-regulation strategies, coping with suicidal ideation and urges) in suicidal individuals as dispositional or situational moderators with regard to STBs. Furthermore, integrating situational variables likely to deplete SSC such as fatigue, stress, and alcohol use into EMA studies would allow for investigating the influence of those situational factors on SSC, emotion-regulation strategies and suicide-specific coping behavior.

Fourth, evidence is limited regarding the associations between implicit suicide-related biases and different self-regulatory mechanisms in relation to the emergence of suicidal behavior. EMA studies should integrate implicit assessments tools to examine the interplay between implicit suicidal markers and processes of the reflective system with reference to suicidal ideation, urges, and behavior. From the perspective of the DSMS, it can be hypothesized that individuals presenting with suicide-specific implicit biases might demonstrate an implicit “preparedness” to react automatically in accordance with these implicit associative patterns under conditions where the reflective system is impaired (e.g., when self-regulatory resources are limited), and/or a suicidal self-control conflict is present. This means that individuals with stronger suicide-specific implicit biases might be less able to restrain suicidal urges and more likely to experience a self-control failure in the form of a suicide attempt, an assumption that has already partially been confirmed (67). Furthermore, implicit associations with death and suicide might facilitate maladaptive suicide-specific coping behavior (e.g., alcohol consumption, social withdrawal), and prevent the use of adaptive coping behavior. Therefore, suicide-specific implicit biases could mediate the association between maladaptive coping and risk for STBs.

Assessing these processes in real time with EMAs may advance the precision of predictive models of suicidal behavior. Besides the many advantages of EMAs, brief mention must also be made of the challenges associated with this methodological approach. While some are more general to all EMA research (e.g., strategies for fostering compliance, the use of a reliable and valid user-friendly app to collect EMA data, careful adherence to current data protection laws, reactivity effects), several are specific to suicide research. First, the relatively low base-rate of suicidal behavior in non-clinical as well as clinical samples should be carefully considered when designing studies as the frequency of the outcome of interest greatly affects sample size and power issues. Second, even though available studies have not observed an iatrogenic effect from assessing suicidality (84, 85), the potential burden of frequently assessing STBs and several related processes over a couple of weeks should be balanced against the expected gain of knowledge by the researchers. Third, the ability and willingness of participants to respond to EMAs during states of affective, cognitive or physiological dysregulation poses a great challenge to research in this area. If self-regulatory resources are limited, study participation (e.g., carrying a smartphone and answering EMA prompts in time) might be seriously affected. In fact, one EMA study demonstrated that non-response was one of the strongest variables in predicting post-hospitalization suicide attempts (22).

Taking these challenges into account, the DSMS facilitates the examination of individual suicidal pathways, covering different suicidal subtypes with different dynamics of the investigated processes, all of which can be theoretically integrated into the DSMS. Assumptions of simple pathways get replaced by a fine-grained analysis of the interaction between reflective and impulsive processes and situational moderators. Moreover, the DSMS represents a *basic framework*, which can be extended with further variables that fit into it, such as control motivation (86) or negative urgency (87). Furthermore, related theoretical approaches like the dynamical systems theory, catastrophe theory (10) or the mindsponge theory (9) can be integrated into the DSMS, because the model incorporates the proposed non-linear processes and leaves room for theoretical collaborations and expansions.

## Conclusion and clinical implications

The present review has focused on the idea that suicidal individuals do not exclusively experience STBs on a continuum as is proposed by many ideation-to-action models of suicidality (4). Growing evidence suggests that suicide risk highly fluctuates within a person, and risk factors substantially differ between persons, a fact that is reflected in the variety of suicidal courses that play out from one individual to the next. Therefore, we need a theoretical framework which is more flexible and integrates a wide range of basic psychological processes that lead to STBs. We argue that suicide research has much to learn from related disciplines like social psychology, which have developed elaborated models of human functioning such as the dual-system framework of information processing. In our view, dual-system frameworks provide a more comprehensive view of human information-processing and behavior and are therefore well-suited for use in suicide research. With the Dual-System Model of Suicidality, we suggest a basic framework based on the idea that two different systems of information-processing, the reflective and the impulsive system, are involved in the development of different STB pathways. The model integrates a great amount of existing suicide research evidence without claiming to be exhaustive, thus leaving room for the investigation and integration of further relevant variables into the DSMS. In the following material, we want to summarize the most important assumptions of the DSMS and discuss implications for clinical practice.

### Maladaptive self-regulation as a vulnerability for suicidal thoughts and behaviors

At the beginning of this paper, we outlined how STBs can be conceptualized from a feedback-based view, which represents the reflective system of information processing. Consistent with this feedback-based view is the assumption that suicidal thoughts, plans and behaviors can be viewed as goal-directed processes controlled from the top-down in order to cope (albeit in maladaptive way) with an aversive situation caused by a discrepancy between where a person perceives themselves as being and where they want to be in terms of important personal goals and standards. We further touched on how a feedback-based view can help to identify dysfunctional self-regulatory processes that facilitate the development of aversive negative affect and states of acute stress. According to the DSMS, *dysfunctional or unattainable goals* may represent one source of persistent discrepancies between actual and desired states that result in perceived incongruence. If an individual does not have adequate problem-solving skills and/or emotion-regulation skills, then incongruence, negative affect, and high levels of stress remain or even increase, creating a vicious circle. Therefore, an individual’s maladaptive self-regulation ability can be viewed as a *vulnerability* for experiencing highly aversive affective states and developing STBs.

### Planned suicidal behavior as maladaptive self-regulatory coping behavior

For some individuals, this can lead to consciously controlled processes whereby they actively cope with this situation by deliberately planning and making a suicide attempt in what could be a reasoned action, an idea that is in line with recent ideation-to-action models of suicidality (e.g., ITS; IMVM). That said, we know little about the underlying self-regulation processes of planned suicidal pathways and how these processes differ compared to impulsive suicidal pathways. Studies have shown that planned suicidal attempts were associated with a greater intent to die and were more lethal compared to impulsive suicide attempts (11), something which might indicate that it represents an action of the reflective system controlled by top-down executive functions. From the perspective of the DSMS, it can be further hypothesized, that planned suicide attempts should be rather independent of emotionally charged situations and should be less associated with momentary states of stress, affective and cognitive dysregulation, and alcohol use, an idea that is supported by the findings of several studies (11, 77, 78). Planned suicide attempts might rather reflect the final result of a process characterized by the existence of feelings of failure and a general low self-efficacy regarding one’s own problem-solving skills persisting with only slight temporal fluctuations. With regard to motivational ambivalence, it can be further speculated that planned suicide attempters should perceive fewer goal conflicts with regard to killing themselves, and experience a more stable wish to die, with self-control conflicts playing a subordinate role compared to impulsive suicide attempters. Research about suicidal phenotypes supports this view (11), showing that not all suicidal pathways reflect a diathesis-stress-model in which suicide attempts represent a reaction to stressful life events.

### Impulsive processes facilitating suicidal thoughts and behaviors

In addition to this feedback-based view of STBs and its multiple implications for the development of STBs, we also introduced a second system of information processing, the impulsive system. We emphasized that the impulsive system can be associated with bottom-up, self-organized processes that operate quickly without any top-down control. From this self-organizing perspective, STBs can also be viewed as processes that emerge in a person without their ever having the conscious goal of killing themself. Rather, it can be viewed as a self-organizing pattern of the impulsive system that is activated to reduce existing incongruence and related highly aversive tension in the system when the reflective system is not able to function anymore. We have further outlined that implicit suicide-specific biases reflect associative patterns of the impulsive system at the unconscious level, which influence STBs. Furthermore, some suicidal individuals experience states of affective, cognitive, and physical dysregulation, which can be viewed as suicidal impulsive precursors emerging from self-organizing processes of the impulsive system that are unintentional and self-reinforcing and therefore cannot be viewed as processes of the reflective system. Those suicidal impulsive precursors signal that the influence of the reflective system is severely limited and that, due to limited cognitive and control resources, an individual is no longer capable of higher-order mental operations such as long-term goals settings, self-monitoring, or exercising restraint in accordance with important long-term goals. These situations represent high-risk states, in which self-regulation can completely break down and self-regulation failure can occur in the form of an impulsive suicide attempt.

### Limited reflective system resources for coping with stress, negative affect, and suicidal urges

With regard to reflective system resources, the DSMS integrates assumptions of the strength model of self-regulation (50) in order to identify risk factors and high risk situations in which the reflective system’s influence is hampered, making way for the impulsive system to gain the upper hand and increase the likelihood of a suicide attempt. In this context, we have introduced the construct of SSC which we have defined as an individual’s momentary capacity to actively control or suppress suicidal urges and impulses. In our view, this variable is highly relevant to suicide research because it reflects a person’s ability to control suicidal urges in the moment and makes it possible to identify high-risk situations in which SSC is low and the likelihood of a suicide attempt in the form of a self-control failure is high. Furthermore, by integrating the concept of limited self-regulation resources and self-control capacity, it becomes possible to investigate associated variables that deplete these resources and facilitate maladaptive impulsive processes.

In this review, we have addressed several conditions under which self-regulation resources can become depleted. A *maladaptive self-regulation per se* on the different levels of the feedback loop can increase a person’s *vulnerability to diminished self-regulation resources* as dysfunctional self-regulation engenders the need for further self-regulation, making it more likely that reflective system resources wane. Maladaptive self-regulation can lead to some individuals experiencing *suicidal impulsive precursors* which are highly stressful and further deplete their self-regulatory resources. Additionally, some individuals experience motivational ambivalence in relation to suicidal behavior and are thus subject to *a self-control conflict* when they have suicidal thoughts and urges. Controlling those suicidal thoughts, urges, and behaviors then consumes self-regulation resources and contributes to a state of ego depletion. Additionally, such states are often accompanied by *situational factors* such as sleep problems and fatigue that further reduce momentary self-regulatory resources. Due to this taxing situation, substance use represents a common coping strategy for dealing with those aversive states (47), all the while acting as a further risk factor draining a person’s reflective system resources.

With regard to more impulsive suicidal pathways, the reflective and impulsive processes described by the DSMS can be considered a *diatheses-stress-model*, according to which poor self-regulation represents a diathesis for degraded self-regulatory resources and the development of suicide-related impulsive processes. Due to additional situational factors that further deplete momentary self-regulation resources, an individual’s *critical threshold* can be exceeded, leading to a self-regulation collapse and potential impulsive suicide attempt.

### Implications for clinical practice

From a clinical perspective, more fine-grained analysis of the underlying processes of STBs supports the development of tailored interventions targeting all three domains of the DSMS: maladaptive self-regulatory and impulsive processes as well as reinforcing situational factors. With regard to dysfunctional self-regulation, changing the goal structure of suicidal patients by implementing higher-order goals, replacing avoidance goals (e.g., avoid failure) with approach goals (e.g., meet friends), and supporting goal attainment (e.g., support disengagement from unattainable goals and re-engagement with more adaptive goals) are critical targets for reducing incongruence and, by extension, a person’s vulnerability to developing STBs. Furthermore, facilitating adaptive strategies for coping with negative life events (e.g., problem-solving therapy (88, 89), negative emotions, stress, and suicidal urges are all forms of interventions that target the reflective system by improving self-regulatory abilities. Such strategies would also condition impulsive processes by reducing the occurrence of suicidal impulsive precursors such as states of high agitation and affective and cognitive dysregulation. As a result, a person’s self-regulatory resources would be protected from rapid or chronic depletion, their capacity for self-control could increase, and they would be empowered to more successfully resist future suicidal urges in high-risk situations.

Furthermore, interventions focus on adaptive suicide-specific coping behavior might simultaneously change implicit attitudes and automatic approach tendencies related to suicide and thus reduce maladaptive impulsive processes. Additionally, direct interventions designed to change implicit cognitive biases or facilitate inhibitory control regarding suicidal urges might decrease the likelihood of a self-control failure, as has been successfully demonstrated by interventions with substance abuse disorders (90). Finally, interventions targeting moderator variables that further deplete self-control and reinforce impulsive processes, such as fatigue, acute stress, and alcohol consumption are essential in order to individuals avoid exceeding the threshold for self-control failures. Therefore, treating sleep disturbances and substance abuse disorders as well as teaching stress management skills are highly recommended for improving adaptive self-regulation and managing future suicidal urges.

What is more, the DSMS has the potential to provide patients and clinicians with an intuitive framework for better understanding the underlying processes that lead to an individual’s suicidal crisis. For clinicians, it could be important to know that in the presence of suicidal pathways dominated by the reflective system, that is, planned suicide attempts, the constellation of risk factors, processes, and situations might substantially differ compared to impulsive suicidal pathways. As we have outlined above, planned suicidal pathways might be less associated with states of affective and cognitive dysregulation, motivational ambivalence, and self-control conflicts. By contrast, impulsive suicidal pathways reflect a diathesis-stress-association whereby the impulsive system controls information-processing and behavior via maladaptive suicidal impulses when an individual’s stress level increases.

Furthermore, both clinicians and patients need to understand that everyone’s self-regulation resources are limited and can be quickly exhausted under several circumstances. They should be aware that in such high-risk situations, impulsive processes can gain the upper hand, and suicidal behavior can (suddenly) emerge as a form of self-organization to reduce overall error in the system, independent of suicidal thoughts, desire, or intent in the moment. Once the pattern has been established, even a small correspondence to the pattern can activate the whole pattern. Therefore, it is crucial to analyze the individual dynamics of factors proposed by the DSMS under which STBs occur. Depending on the suicidal pattern, tailored interventions should target dominant factors and processes individually. For example, if a person shows a strong wish to die regardless of personal long-term goals, interventions should focus on the level of goals they have set for themselves, related goal conflicts, and ambivalence with techniques such as motivational interviewing (91) or cognitive restructuring (92). Such interventions might simultaneously change implicit attitudes toward death and lead to more adaptive coping behavior regarding existing incongruences. If, on the other hand, impulsive processes such as high anxiety and agitation are dominant, stress-reducing and emotion-regulation skills should be the focus of treatment, with the goals of avoiding chronic ego depletion and strengthening the person’s self-regulation capacity in the long-term.

Some of the proposed interventions have already been implemented in the practice guidelines for the treatment of STBs (93), progress that can be built on in suicide research by utilizing the framework of the DSMS to investigate the underlying within-person change mechanisms, in part by determining the effectiveness of certain interventions in reducing suicide risk as well as by potentially identifying other processes or components to target. Despite its limitations, using EMA as a technique for assessing these processes in real time could advance the precision of predictive models of suicidal behavior and lead to more refined interventions, the success of which could likewise be measured with EMA.

## Author contributions

JB conceptualized and wrote the manuscript. LS drafted and revised the manuscript. HG and TB read and contributed to manuscript revision. All authors reviewed and approved the final draft.

## References

[1] World Health Organization [WHO]. *Suicide.* (2019). Available online at: http://www.who.int/mediacentre/factsheets/fs398/en/ (Accessed September 28, 2022).

[2] Large M Kaneson M Myles N Myles H Gunaratne P Ryan C. Meta-analysis of longitudinal cohort studies of suicide risk assessment among psychiatric patients: heterogeneity in results and lack of improvement over time. *PLoS One.* (2016) 11:e0156322. 10.1371/journal.pone.0156322 27285387 PMC4902221

[3] Franklin JC Ribeiro JD Fox KR Bentley KH Kleiman EM Huang X Risk factors for suicidal thoughts and behaviors: a meta-analysis of 50 years of research. *Psychol Bull.* (2017) 143:187–232. 10.1037/bul0000084 27841450

[4] Klonsky ED Qiu T Saffer BY. Recent advances in differentiating suicide attempters from suicide ideators. *Curr Opin Psychiatry.* (2017) 30:15–20. 10.1097/YCO.0000000000000294 27798483

[5] O’Connor RC Nock MK. The psychology of suicidal behaviour. *Lancet Psychiatry.* (2014) 1:73–85. 10.1016/S2215-0366(14)70222-6 26360404

[6] van Orden KA Witte TK Cukrowicz KC Braithwaite SR Selby EA Joiner TE. The interpersonal theory of suicide. *Psychol Rev.* (2010) 117:575–600. 10.1037/a0018697 20438238 PMC3130348

[7] O’Connor RC Kirtley OJ. The integrated motivational–volitional model of suicidal behaviour. *Phil Trans R Soc B.* (2018) 373:20170268. 10.1098/rstb.2017.0268 30012735 PMC6053985

[8] Millner AJ Robinaugh DJ Nock MK. Advancing the understanding of suicide: the need for formal theory and rigorous descriptive research. *Trends Cogn Sci.* (2020) 24:704–16. 10.1016/j.tics.2020.06.007 32680678 PMC7429350

[9] Nguyen M-H Le T-T Nguyen H-KT Ho M-T Nguyen HT Vuong Q-H. Alice in suicideland: exploring the suicidal ideation mechanism through the sense of connectedness and help-seeking behaviors. *Int J Environ Res Public Health.* (2021) 18:3681. 10.3390/ijerph18073681 33916123 PMC8037954

[10] Bryan CJ Rudd MD. The importance of temporal dynamics in the transition from suicidal thought to behavior. *Clin Psychol.* (2016) 23:21–5. 10.1111/cpsp.12135

[11] Bernanke JA Stanley BH Oquendo MA. Toward fine-grained phenotyping of suicidal behavior: the role of suicidal subtypes. *Mol Psychiatry.* (2017) 22:1080–1. 10.1038/mp.2017.123 28607457 PMC5785781

[12] Wastler HM Bryan AO Bryan CJ. Suicide attempts among adults denying active suicidal ideation: an examination of the relationship between suicidal thought content and suicidal behavior. *J Clin Psychol.* (2022) 78:1103–17. 10.1002/jclp.23268 34674388

[13] Wyder M DeLeo D. Behind impulsive suicide attempts: indications from a community study. *J Affect Disord.* (2007) 104:167–73. 10.1016/j.jad.2007.02.015 17397934

[14] Hallensleben N Spangenberg L Forkmann T Rath D Hegerl U Kersting A Investigating the dynamics of suicidal ideation. *Crisis.* (2018) 39:65–9. 10.1027/0227-5910/a000464 28468557

[15] Kleiman EM Turner BJ Fedor S Beale EE Huffman JC Nock MK. Examination of real-time fluctuations in suicidal ideation and its risk factors: results from two ecological momentary assessment studies. *J Abnorm Psychol.* (2017) 126:726–38. 10.1037/abn0000273 28481571

[16] Brüdern J Berger T Gysin Maillart A Michel K Caspar F. The role of self-organization in the suicidal process. *Psychol Rep.* (2016) 118:668–85. 10.1177/0033294116633351 27154385

[17] Millner AJ Lee MD Nock MK. Single-item measurement of suicidal behaviors: validity and consequences of misclassification. *PLoS One.* (2015) 10:e0141606. 10.1371/journal.pone.0141606 26496707 PMC4619664

[18] Yaseen ZS Hawes M Barzilay S Galynker I. Predictive validity of proposed diagnostic criteria for the suicide crisis syndrome: an acute presuicidal state. *Suicide Life Threat Behav.* (2019) 49:1124–35. 10.1111/sltb.12495 30073686

[19] Ballard ED Gilbert JR Wusinich C Zarate CA. New methods for assessing rapid changes in suicide risk. *Front Psychiatry.* (2021) 12:598434. 10.3389/fpsyt.2021.598434 33574775 PMC7870718

[20] Ammerman B. Using intensive time sampling methods to capture daily suicidal ideation: a systematic review. *J Affect Disord.* (2021) 299:108–17. 10.1016/j.jad.2021.10.121 34718039

[21] Trull TJ Ebner-Priemer UW. Ambulatory assessment in psychopathology research: a review of recommended reporting guidelines and current practices. *J Abnorm Psychol.* (2020) 129:56–63. 10.1037/abn0000473 31868388

[22] Wang SB Coppersmith DD Kleiman EM Bentley KH Millner AJ Fortgang R A pilot study using frequent inpatient assessments of suicidal thinking to predict short-term postdischarge suicidal behavior. *JAMA Netw Open.* (2021) 4:e210591. 10.1001/jamanetworkopen.2021.0591 33687442 PMC7944382

[23] Steeg S Quinlivan L Nowland R Caroll R Casey D Clements C Accuracy of risk scales for predicting repeat self-harm and suicide: a multicentre, population-level cohort study using routine clinical data. *BMC Psychiatry.* (2018) 18:113. 10.1186/s12888-018-1693-z 29699523 PMC5921289

[24] NICE guideline [NG225]. *Self-Harm: Assessment, Management and Preventing Recurrence.* (2022). Available online at: https://www.nice.org.uk/guidance/ng225 (Accessed September 28, 2022). 36595613

[25] Epstein S Pacini R Denes-Raj V Heier H. Individual differences in intuitive-experiential and analytical-rational thinking styles. *J Pers Soc Psychol.* (1996) 71:390–405. 10.1037//0022-3514.71.2.390 8765488

[26] Carver CS Scheier MF. Control processes and self-organization as complementary principles underlying behavior. *Pers Soc Psychol Rev.* (2002) 6:304–15.

[27] Strack F Deutsch R. Reflective and impulsive determinants of social behavior. *Pers Soc Psychol Rev.* (2004) 8:220–47. 15454347 10.1207/s15327957pspr0803_1

[28] Metcalfe J Mischel W. A hot/cool-system analysis of delay of gratification: dynamics of willpower. *Psychol Rev.* (1999) 106:3–19. 10.1037/0033-295x.106.1.3 10197361

[29] Chu C Buchman-Schmitt JM Stanley IH Hom MA Tucker RP Hagan CR The interpersonal theory of suicide: a systematic review and meta-analysis of a decade of cross-national research. *Psychol Bull.* (2017) 143:1313–45. 10.1037/bul0000123 29072480 PMC5730496

[30] Hallensleben N Glaesmer H Forkmann T Rath D Strauss M Kersting A Predicting suicidal ideation by interpersonal variables, hopelessness and depression in real-time. An ecological momentary assessment study in psychiatric inpatients with depression. *Eur Psychiatry.* (2019) 56:43–50. 10.1016/j.eurpsy.2018.11.003 30530103

[31] Kuehn KS Dora J Harned MS Foster KT Song F Smith MR A meta-analysis on the affect regulation function of real-time self-injurious thoughts and behaviours. *Nat Hum Behav.* (2022) 6:964–74. 10.1038/s41562-022-01340-8 35484208 PMC9329197

[32] Vohs KD Baumeister RF. Escaping the self consumes regulatory resources: a self-regulatory model of suicide. In: Joiner T Rudd MD editors. *Suicide Science: Expanding the Boundaries.* Norwell, MA: Kluwer Academic Publishers (2000). p. 33–41.

[33] Michalak J Grosse Holtforth M. Where do we go from here? The goal perspective in psychotherapy. *Clin Psychol.* (2006) 13:346–65.

[34] Ma J Batterham PJ Calear AL Han J. A systematic review of the predictions of the interpersonal-psychological theory of suicidal behavior. *Clin Psychol Rev.* (2016) 46:34–45. 10.1016/j.cpr.2016.04.008 27155061

[35] O’Connor RC Portzky G. The relationship between entrapment and suicidal behavior through the lens of the integrated motivational-volitional model of suicidal behavior. *Curr Opin Psychol.* (2018) 22:12–7. 10.1016/j.copsyc.2017.07.021 30122271

[36] O’Connor RC Smyth R Ferguson E Ryan C Williams JM. Psychological processes and repeat suicidal behavior: a four-year prospective study. *J Consult Clin Psychol.* (2013) 81:1137–43. 10.1037/a0033751 23855989 PMC3933214

[37] Brüdern J Berger T Michel K Maillart AG Held IS Caspar F. Are suicide attempters wired differently? A comparison with nonsuicidal depressed individuals using plan analysis. *J Nerv Ment Dis.* (2015) 203:514–21. 10.1097/NMD.0000000000000321 26057773

[38] O’Connor RC. The relations between perfectionism and suicidality: a systematic review. *Suicide Life Threat Behav.* (2007) 37:698–714. 10.1521/suli.2007.37.6.698 18275376

[39] Sommerfeld E Malek S. Perfectionism moderates the relationship between thwarted belongingness and perceived burdensomeness and suicide ideation in adolescents. *Psychiatr Q.* (2019) 90:671–81. 10.1007/s11126-019-09639-y 31037588

[40] O’Connor RC O’Carroll RE Ryan C Smyth R. Self-regulation of unattainable goals in suicide attempters: a two year prospective study. *J Affect Disord.* (2012) 142:248–55. 10.1016/j.jad.2012.04.035 22980400

[41] Vallacher RR Wegner DM. What do people think they’re doing? Action identification and human behavior. *Psychol Rev.* (1987) 94:3–15.

[42] Richardson C Dickson A Robb KA O’Connor RC. The male experience of suicide attempts and recovery: an interpretative phenomenological analysis. *Int J Environ Res Public Health.* (2021) 18:5209. 10.3390/ijerph18105209 34068854 PMC8153566

[43] Horwitz AG Czyz EK Berona J King CA. Prospective associations of coping styles with depression and suicide risk among psychiatric emergency patients. *Behav Ther.* (2018) 49:225–36. 10.1016/j.beth.2017.07.010 29530261

[44] Gysin-Maillart A Soravia L Schwab S. Attempted suicide short intervention program influences coping among patients with a history of attempted suicide. *J Affect Disord.* (2020) 264:393–9. 10.1016/j.jad.2019.11.059 31759660

[45] Hallard RI Wells A Aadahl V Emsley R Pratt D. Metacognition, rumination and suicidal ideation: an experience sampling test of the self-regulatory executive function model. *Psychiatry Res.* (2021) 303:114083. 10.1016/j.psychres.2021.114083 34271370

[46] Czyz EK Glenn CR Busby D King CA. Daily patterns in nonsuicidal self-injury and coping among recently hospitalized youth at risk for suicide. *Psychiatry Res.* (2019) 281:112588. 10.1016/j.psychres.2019.112588 31629299 PMC6890202

[47] Simon GE Specht C Doederlein A. Coping with suicidal thoughts: a survey of personal experience. *Psychiatr Serv.* (2016) 67:1026–9. 10.1176/appi.ps.201500281 27247170 PMC5008973

[48] Stanley B Martínez-Alés G Gratch I Rizk M Galfalvy H Choo T Coping strategies that reduce suicidal ideation: an ecological momentary assessment study. *J Psychiatr Res.* (2021) 133:32–7. 10.1016/j.jpsychires.2020.12.012 33307352 PMC8659118

[49] Al-Dajani N Horwitz AG Czyz EK. Does coping reduce suicidal urges in everyday life? Evidence from a daily diary study of adolescent inpatients. *Depress Anxiety.* (2022) 39:496–503. 10.1002/da.23253 35322919 PMC9246857

[50] Muraven M Baumeister RF. Self-regulation and depletion of limited resources: does self-control resemble a muscle? *Psychol Bull.* (2000) 126:247–59. 10.1037/0033-2909.126.2.247 10748642

[51] Baumeister RF. Ego depletion and self-regulation failure: a resource model of self-control. *Alcohol Clin Exp Res.* (2003) 27:281–4. 10.1097/01.ALC.0000060879.61384.A4 12605077

[52] Dang J Barker P Baumert A Bentvelzen M Berkman E Buchholz N A multilab replication of the ego depletion effect. *Soc Psychol Personal Sci.* (2021) 12:14–24. 10.1177/1948550619887702 34113424 PMC8186735

[53] Muraven M Collins RL Nienhaus K. Self-control and alcohol restraint: an initial application of the self-control strength model. *Psychol Addict Behav.* (2002) 16:113–20. 10.1037//0893-164x.16.2.113 12079249

[54] Bryan CJ Butner JE May AM Rugo KF Harris J Oakey DN Nonlinear change processes and the emergence of suicidal behavior: a conceptual model based on the fluid vulnerability theory of suicide. *New Ideas Psychol.* (2020) 57:100758. 10.1016/j.newideapsych.2019.100758 32123464 PMC7050543

[55] Carver CS Scheier MF. Self-regulation of action and affect. In: Vohs KD Baumeister RF editors. *Handbook of Self-Regulation.* New York, NY: Guilford Press (2004). p. 3–23.

[56] Tangney JP Baumeister RF Boone AL. High self-control predicts good adjustment, less pathology, better grades, and interpersonal success. *J Pers.* (2004) 72:271–324. 10.1111/j.0022-3506.2004.00263.x 15016066

[57] Betrams A Unger A Dickhäuser O. Momentan verfügbare Selbstkontrollkraft - Vorstellung eines Messinstruments und erste Befunde aus pädagogisch-psychologischen Kontexten [Momentarily available self-control strength – introduction of a measure and first findings from educational-psychological contexts]. *Zeitschrift Pädagog Psychol.* (2011) 25:185–96.

[58] Brown GK Steer RA Henriques GR Beck AT. The internal struggle between the wish to die and the wish to live: a risk factor for suicide. *Am J Psychiatry.* (2005) 162:1977–9. 10.1176/appi.ajp.162.10.1977 16199851

[59] Brüdern J Stähli A Gysin-Maillart A Michel K Reisch T Jobes DA Reasons for living and dying in suicide attempters: a two-year prospective study. *BMC Psychiatry.* (2018) 18:234. 10.1186/s12888-018-1814-8 30029631 PMC6053763

[60] Hofmann W Friese M Strack F. Impulse and Self-Control From a Dual-Systems Perspective. *Perspect Psychol Sci.* (2009) 4:162–76. 10.1111/j.1745-6924.2009.01116.x 26158943

[61] Hofmann W Friese M Wiers RW. Impulsive versus reflective influences on health behavior: a theoretical framework and empirical review. *Health Psychol Rev.* (2008) 2:111–37. 10.1080/17437190802617668

[62] Carver CS Scheier MF. *On the Self-Regulation of Behavior.* Cambridge: Cambridge University Press (2001). p. 439.

[63] Rath D de Beurs D Hallensleben N Spangenberg L Glaesmer H Forkmann T. Predicting suicide ideation from beep to beep: application of network analysis to ecological momentary assessment data. *Internet Interv.* (2019) 18:100292. 10.1016/j.invent.2019.100292 31828015 PMC6889482

[64] Robinaugh DJ Hoekstra RHA Toner ER Borsboom D. The network approach to psychopathology: a review of the literature 2008–2018 and an agenda for future research. *Psychol Med.* (2020) 50:353–66. 10.1017/S0033291719003404 31875792 PMC7334828

[65] Schiepek G Fartacek C Sturm J Kralovec K Fartacek R Plöderl M. Nonlinear dynamics: theoretical perspectives and application to suicidology. *Suicide Life Threat Behav.* (2011) 41:661–75. 10.1111/j.1943-278X.2011.00062.x 22145825

[66] Nock MK Park JM Finn CT Deliberto TL Dour HJ Banaji MR. Measuring the suicidal mind: implicit cognition predicts suicidal behavior. *Psychol Sci.* (2010) 21:511–7. 10.1177/0956797610364762 20424092 PMC5258199

[67] Moreno M Gutiérrez-Rojas L Porras-Segovia A. Implicit cognition tests for the assessment of suicide risk: a systematic review. *Curr Psychiatry Rep.* (2022) 24:141–59. 10.1007/s11920-022-01316-5 35150387 PMC8852938

[68] Cha CB Najmi S Park JM Finn CT Nock MK. Attentional bias toward suicide-related stimuli predicts suicidal behavior. *J Abnorm Psychol.* (2010) 119:616–22. 10.1037/a0019710 20677851 PMC2994414

[69] Wilson KM Millner AJ Auerbach RP Glenn CR Kearns JC Kirtley OJ Investigating the psychometric properties of the suicide stroop task. *Psychol Assess.* (2019) 31:1052–61. 10.1037/pas0000723 31070448 PMC7011179

[70] Galynker I Yaseen ZS Cohen A Benhamou O Hawes M Briggs J. Prediction of suicidal behavior in high risk psychiatric patients using an assessment of acute suicidal state: the suicide crisis inventory. *Depress Anxiety.* (2017) 34:147–58. 10.1002/da.22559 27712028

[71] Spangenberg L Forkmann T Glaesmer H. *APOS: Acute Risk Factors for Post-Discharge Suicidal Behavior.* (2022). Available online at: https://archive.org/details/osf-registrations-axnws-v1 (Accessed September 28, 2022).

[72] Hagger MS Wood C Stiff C Chatzisarantis NL. Ego depletion and the strength model of self-control: a meta-analysis. *Psychol Bull.* (2010) 136:495–525. 10.1037/a0019486 20565167

[73] Diestel S Rivkin W Schmidt K-H. Sleep quality and self-control capacity as protective resources in the daily emotional labor process: results from two diary studies. *J Appl Psychol.* (2015) 100:809–27. 10.1037/a0038373 25486259

[74] Brüdern J Hallensleben N Höller I Spangenberg L Forkmann T Rath D Sleep disturbances predict active suicidal ideation the next day: an ecological momentary assessment study. *BMC Psychiatry.* (2022) 22:65. 10.1186/s12888-022-03716-6 35086519 PMC8793208

[75] Hamilton JL Tsypes A Zelazny J Sewall CJ Rode N Merranko J Sleep influences daily suicidal ideation through affective reactivity to interpersonal events among high-risk adolescents and young adults. *J Child Psychol Psychiatry.* (2022). [Epub ahead of print]. 10.1111/jcpp.13651 35778912 PMC9876533

[76] Kleiman EM Turner BJ Chapman AL Nock MK. Fatigue moderates the relationship between perceived stress and suicidal ideation: evidence from two high-resolution studies. *J Clin Child Adolesc Psychol.* (2018) 47:116–30. 10.1080/15374416.2017.1342543 28715280

[77] Kõlves K Koo YW de Leo D. A drink before suicide: analysis of the Queensland Suicide Register in Australia. *Epidemiol Psychiatr Sci.* (2020) 29:e94. 10.1017/S2045796020000062 31973775 PMC7214701

[78] Conner KR Hesselbrock VM Meldrum SC Schuckit MA Bucholz KK Gamble SA Transitions to, and correlates of, suicidal ideation, plans, and unplanned and planned suicide attempts among 3,729 men and women with alcohol dependence. *J Stud Alcohol Drugs.* (2007) 68:654–62. 10.15288/jsad.2007.68.654 17690798

[79] Cherpitel CJ Borges GL Wilcox HC. Acute alcohol use and suicidal behavior: a review of the literature. *Alcohol Clin Exp Res.* (2004) 28:18S–28S. 10.1097/01.alc.0000127411.61634.14 15166633

[80] Bryan CJ Garland EL Rudd MD. From impulse to action among military personnel hospitalized for suicide risk: alcohol consumption and the reported transition from suicidal thought to behavior. *Gen Hosp Psychiatry.* (2016) 41:13–9. 10.1016/j.genhosppsych.2016.05.001 27302719

[81] Cha CB O’Connor RC Kirtley O Cleare S Wetherall K Eschle S Testing mood-activated psychological markers for suicidal ideation. *J Abnorm Psychol.* (2018) 127:448–57. 10.1037/abn0000358 29927267

[82] Bentley KH Coppersmith DL Kleiman EM Nook EC Mair P Millner AJ Do patterns and types of negative affect during hospitalization predict short-term post-discharge suicidal thoughts and behaviors?. *Affect Sci.* (2021) 2:484–94. 10.1007/s42761-021-00058-6 35465415 PMC9022604

[83] Wenzel M Rowland Z Kubiak T. Examining five pathways on how self-control is associated with emotion regulation and affective well-being in daily life. *J Pers.* (2021) 89:451–67. 10.1111/jopy.12590 32924133

[84] Law MK Furr RM Arnold EM Mneimne M Jaquett C Fleeson W. Does assessing suicidality frequently and repeatedly cause harm? A randomized control study. *Psychol Assess.* (2015) 27:1171–81. 10.1037/pas0000118 25894705 PMC4615260

[85] DeCou CR Schumann ME. On the iatrogenic risk of assessing suicidality: a meta-analysis. *Suicide Life Threat Behav.* (2018) 48:531–43. 10.1111/sltb.12368 28678380

[86] Kotabe HP Hofmann W. On integrating the components of self-control. *Perspect Psychol Sci.* (2015) 10:618–38. 10.1177/1745691615593382 26386000

[87] King KM Feil MC Halvorson MA. Negative urgency is correlated with the use of reflexive and disengagement emotion regulation strategies. *Clin Psychol Sci.* (2018) 6:822–34. 10.1177/2167702618785619

[88] Owens D Wright-Hughes A Graham L Blenkiron P Burton K Collinson M Problem-solving therapy rather than treatment as usual for adults after self-harm: a pragmatic, feasibility, randomised controlled trial (the MIDSHIPS trial). *Pilot Feasibility Stud.* (2020) 6:119. 10.1186/s40814-020-00668-0 32832098 PMC7437039

[89] Stewart CD Quinn A Plever S Emmerson B. Comparing cognitive behavior therapy, problem solving therapy, and treatment as usual in a high risk population. *Suicide Life Threat Behav.* (2009) 39:538–47. 10.1521/suli.2009.39.5.538 19929153

[90] Houben K Havermans RC Wiers RW. Learning to dislike alcohol: conditioning negative implicit attitudes toward alcohol and its effect on drinking behavior. *Psychopharmacology.* (2010) 211:79–86. 10.1007/s00213-010-1872-1 20431994 PMC2885295

[91] Hoy J. Motivational interviewing and the transtheoretical model of change: under-explored resources for suicide intervention. *Community Ment Health J.* (2016) 52:559–67. 10.1007/s10597-016-9997-2 26886871

[92] Bryan CJ. Cognitive behavioral therapy for suicide prevention (CBT-SP): implications for meeting standard of care expectations with suicidal patients. *Behav Sci Law.* (2019) 37:247–58. 10.1002/bsl.2411 31119794

[93] Jacobs DG Baldessarini RJ Conwell Y Fawcett JA Horton L Meltzer H *Practice Guideline For the Assessment and Treatment of Patients With Suicidal Behaviors.* Washington, DC: American Psychiatric Association (2010). p. 60.

